# Supplementary material for: Alignment of single-cell trajectory trees with CAPITAL
Source: Nat Commun. 2022 Oct 14;13:5972. doi: 10.1038/s41467-022-33681-3 (PMC9568509; doi:10.1038/s41467-022-33681-3)
Supplement: Supplementary file 1 — Supplementary Information [file 41467_2022_33681_MOESM1_ESM.pdf]

Supplementary Information on "Alignment of single-cell trajectory trees with CAPITAL"

Reiichi Sugihara[1], Yuki Kato[1,2,*], Tomoya Mori[3] and Yukio Kawahara[1,2]

1 Department of RNA Biology and Neuroscience, Graduate School of Medicine, Osaka University, 2-2 Yamada-oka, Suita, Osaka 565-0871, Japan

2 Integrated Frontier Research for Medical Science Division, Institute for Open and Transdisciplinary Research Initiatives (OTRI), Osaka University, 2-2 Yamada-oka, Suita, Osaka 565-0871, Japan

3 Bioinformatics Center, Institute for Chemical Research, Kyoto University, Gokasho, Uji, Kyoto 611-0011, Japan

[*]Correspondence should be addressed to Y. Kato (ykato@rna.med.osaka-u.ac.jp).

Contents

1 Supplementary Notes **3**

2 Supplementary Figures **5**

List of Supplementary Figures

1 UMAP plots of clustering results of an example synthetic dataset 5
2 UMAP plots and dot plots of datasets 1 and 2 computed by CAPITAL 6
3 Comparison of the ability to remove batch effects on datasets 3 and 4 8
4 UMAP plots with respect to marker genes for Setty *et al.*'s data 9
5 A dot plot with respect to marker genes and a trajectory tree of Setty *et al.*'s data . . 10
6 UMAP plots with respect to marker genes for Velten *et al.*'s data 11
7 A dot plot with respect to marker genes and a trajectory tree of Velten *et al.*'s data . . 12
8 Pseudotime kinetics for markers in the human bone marrow cells 13
9 An alignment of the trajectory trees of Setty *et al.*'s data and Paul *et al.*'s data 14
10 UMAP plots with respect to marker genes for Paul *et al.*'s data 15
11 A dot plot with respect to marker genes and a trajectory tree of Paul *et al.*'s data . . . 16
12 Pseudotime kinetics for genes with similar patterns between human and mouse . . . 17
13 Pseudotime kinetics for genes with different patterns between human and mouse . . . 19
14 Heatmaps of enriched terms with similar patterns between human and mouse 22
15 A schematic of the DP recursion for aligning trees 23
16 A schematic of the DP recursion for aligning forests 24

1 Supplementary Notes

Supplementary Note 1

Proposition 1. *CAPITAL runs faster than all-against-all alignment of single lineages if input trajectories have one or more branches.*

Proof. For simplicity, we assume that the clusters, the leaves and the cells in one trajectory tree have the same in number as in the other, denoted by N, L and n, respectively.

CAPITAL first computes a cluster tree alignment in $O(N^2)$ time. Second, diffusion pseudotime of $O(n)$ single cells is calculated per branch with solving the eigenvalue problem [1], resulting in $O(Ln^3)$ time in total. Finally, dynamic time warping on those L single lineages is performed in $O(Ln^2)$ time. Thus, the total running time is evaluated as $O(N^2) + O(Ln^3) + O(Ln^2) = O(Ln^3)$ because $N \ll n$ and $L \ll n$ hold in most cases of single-cell data analysis.

In contrast, the all-against-all alignment of single lineages requires $O(L^2)$ comparisons, each of which takes $O(n^3) + O(n^2)$ steps as discussed above. Hence, it runs in $O(L^2n^3)$ time.

Therefore, CAPITAL can run faster than the naive all-against-all linear alignment by a factor of $O(L)$. □

Supplementary Note 2

Assume that the nodes in tree T_k $(k = 1, 2)$ are numbered by 1 through $|T_k|$ in the postorder fashion. The following pseudocode computes an optimal tree alignment distance between the unordered trees with bounded degrees:

procedure TREE-ALIGNMENT-DISTANCE(T_1, T_2)
  Initialize with $D(\theta, \theta) = 0$.
  for $i = 1$ to $|T_1|$
    Compute

$$D(T_1(i), \theta) = D(F_1(i), \theta) + \gamma(i, \lambda), \quad D(F_1(i), \theta) = \sum_{k=1}^{\mu} D(T_1(i_k), \theta).$$

  for $j = 1$ to $|T_2|$
    Compute

$$D(\theta, T_2(j)) = D(\theta, F_2(j)) + \gamma(\lambda, j), \quad D(\theta, F_2(j)) = \sum_{k=1}^{\nu} D(\theta, T_2(j_k)).$$

  for $i = 1$ to $|T_1|$
    for $j = 1$ to $|T_2|$
      Compute

$$\begin{aligned} & D(T_1(i), T_2(j)) \\ &= \min \begin{cases} D(F_1(i), F_2(j)) + \gamma(i, j), \\ D(T_1(i), \theta) + \min_{1 \le r \le \mu} \{D(T_1(i_r), T_2(j)) - D(T_1(i_r), \theta)\}, \\ D(\theta, T_2(j)) + \min_{1 \le r \le \nu} \{D(T_1(i), T_2(j_r)) - D(\theta, T_2(j_r))\}. \end{cases} \end{aligned}$$

10: Compute

$$D(\mathcal{A},\mathcal{B}) = \min \begin{cases} \min_{T_1(i_p)\in\mathcal{A},T_2(j_q)\in\mathcal{B}} \left\{ D\left(\mathcal{A}-T_1(i_p),\mathcal{B}-T_2(j_q)\right) + D\left(T_1(i_p),T_2(j_q)\right) \right\}, \\ \min_{T_1(i_p)\in\mathcal{A},\mathcal{B}'\subseteq\mathcal{B}} \left\{ D\left(F_1(i_p),\mathcal{B}'\right) + D\left(\mathcal{A}-T_1(i_p),\mathcal{B}-\mathcal{B}'\right) + \gamma(i_p,\lambda) \right\}, \\ \min_{\mathcal{A}'\subseteq\mathcal{A},T_2(j_q)\in\mathcal{B}} \left\{ D\left(\mathcal{A}',F_2(j_q)\right) + D\left(\mathcal{A}-\mathcal{A}',\mathcal{B}-T_2(j_q)\right) + \gamma(\lambda,j_q) \right\}. \end{cases}$$

$D\left(T_1(|T_1|),T_2(|T_2|)\right)$ obtained in the above pseudocode will have an optimal alignment distance between T_1 and T_2. To recover the optimal tree alignment, the traceback procedure starting with $D\left(T_1(|T_1|),T_2(|T_2|)\right)$ is needed, where the calculation path to $D\left(T_1(|T_1|),T_2(|T_2|)\right)$ is recovered with traceback pointers that hold the choice of the minimum operations in the dynamic programming recursions.

2 Supplementary Figures

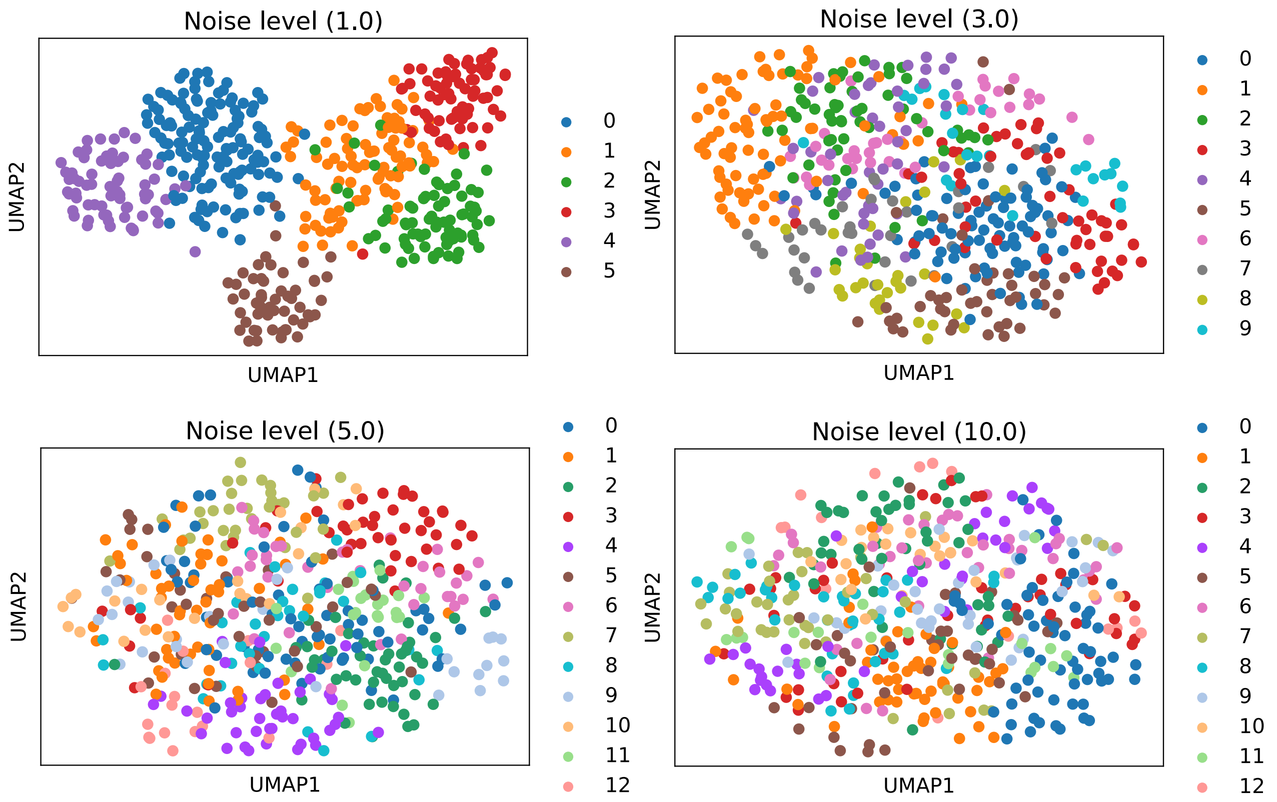

Supplementary Fig. 1 UMAP plots of clustering results of an example synthetic dataset. The number in parentheses that follows each graph title shows the standard deviation of Gaussian noise added to the data. UMAP, uniform manifold approximation and projection.

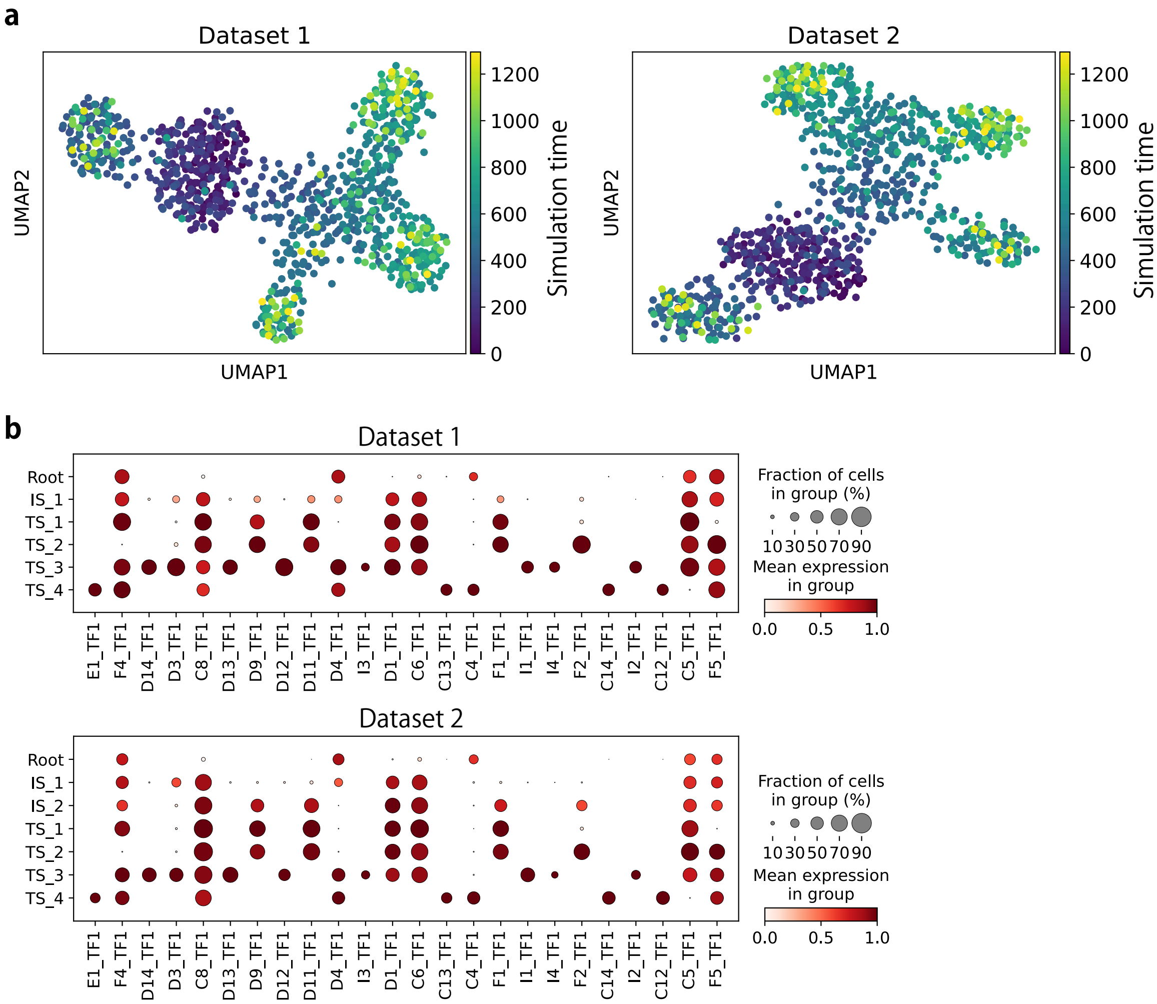

Supplementary Fig. 2 UMAP plots and dot plots of datasets 1 and 2 computed by CAPITAL. **a,** UMAP plots of cells colored by true simulation times on the respective datasets. **b,** Dot plots with respect to the expression levels of transcription factors to validate cluster alignment computed by CAPITAL. UMAP, uniform manifold approximation and projection; IS, intermediate state; TS, terminal state.

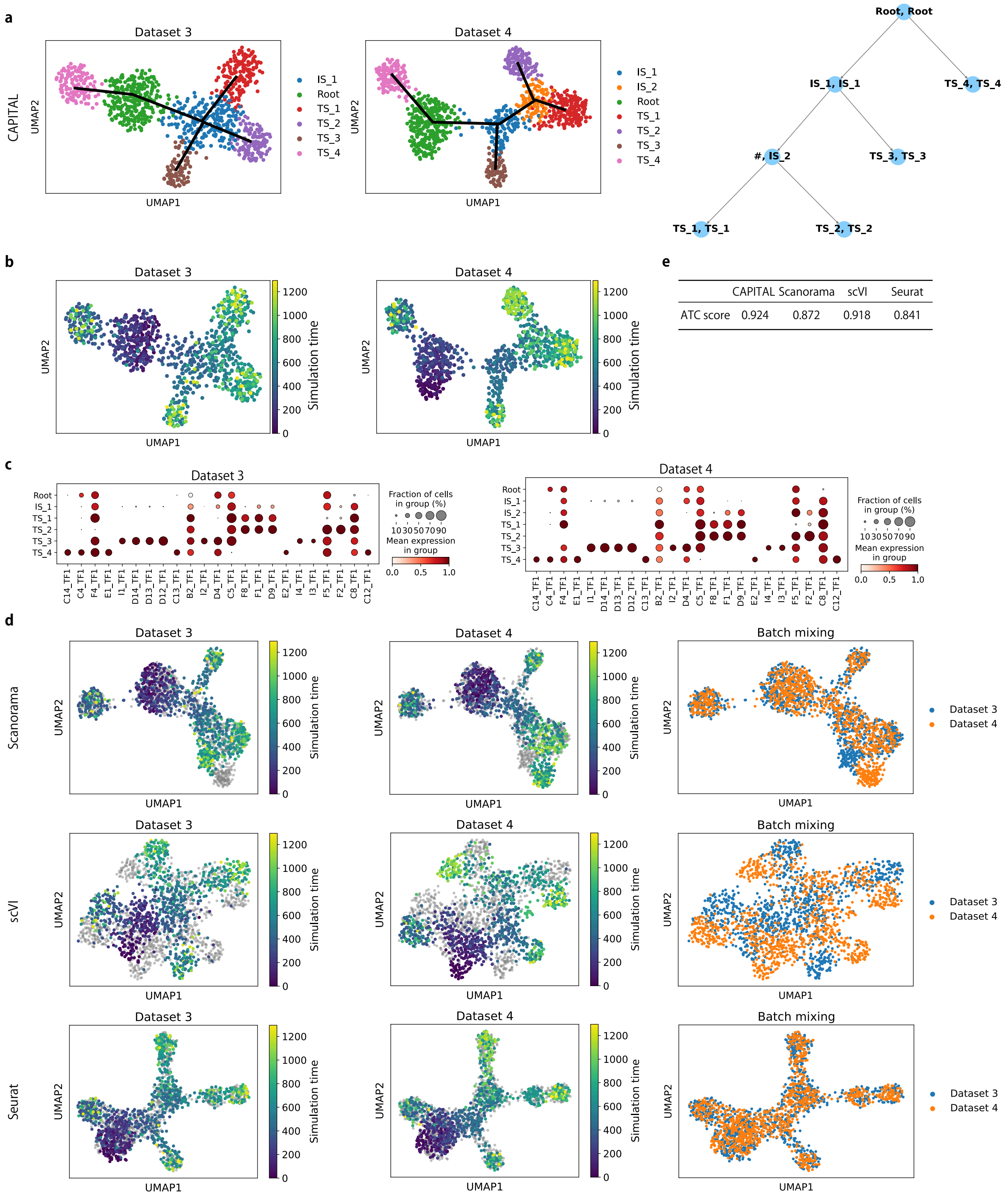

	CAPITAL	Scanorama	scVI	Seurat
ATC score	0.924	0.872	0.918	0.841

Supplementary Fig. 3 Comparison of the ability to remove batch effects between CAPITAL and data integration methods on another pair of the synthetic datasets. **a,** UMAP plots of datasets 3 and 4 with the Leiden clustering, whose cell types were annotated by considering simulation time and expression patterns of transcription factors (this figure b and c). The solid lines indicate the trajectories. The rightmost column shows an aligned trajectory tree of those datasets predicted by CAPITAL. **b,** UMAP plots of cells colored by true simulation times. **c,** Dot plots with respect to the expression levels of transcription factors to validate cluster alignment computed by CAPITAL. **d,** UMAP plots of integration of datasets 3 and 4 computed by three data integration methods. The first and second columns indicate true simulation times in datasets 3 and 4, respectively, on the merged dataset, and the rightmost column shows UMAP plots of batch mixing. **e,** ATC scores of all tools on datasets 3 and 4. UMAP, uniform manifold approximation and projection; IS, intermediate state; TS, terminal state, ATC, average trajectory conservation.

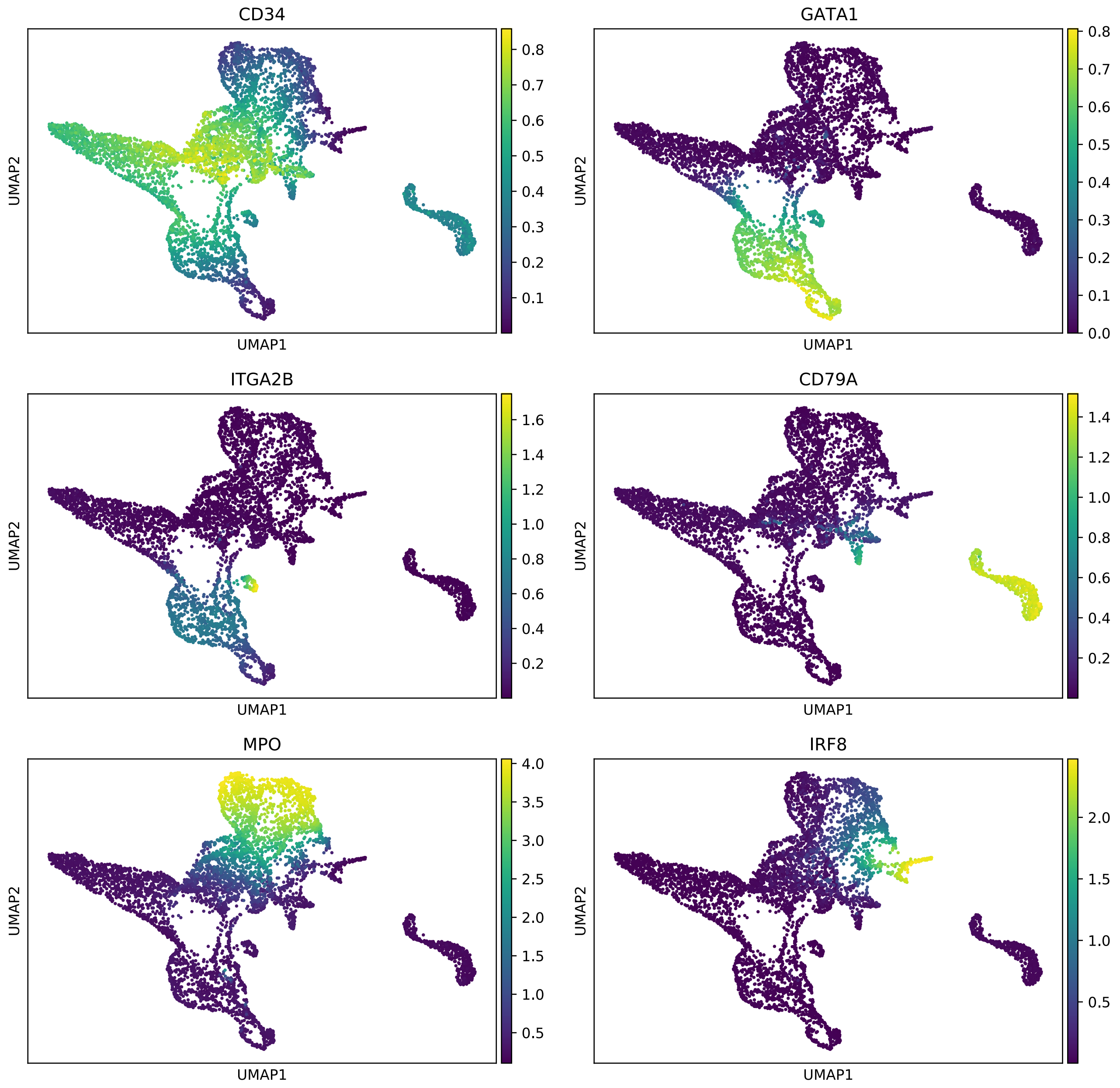

Supplementary Fig. 4 UMAP plots of cells colored by expression levels of marker genes for Setty *et al.*'s data. Selection of marker genes used in this work is based on the literature [2]. UMAP, uniform manifold approximation and projection.

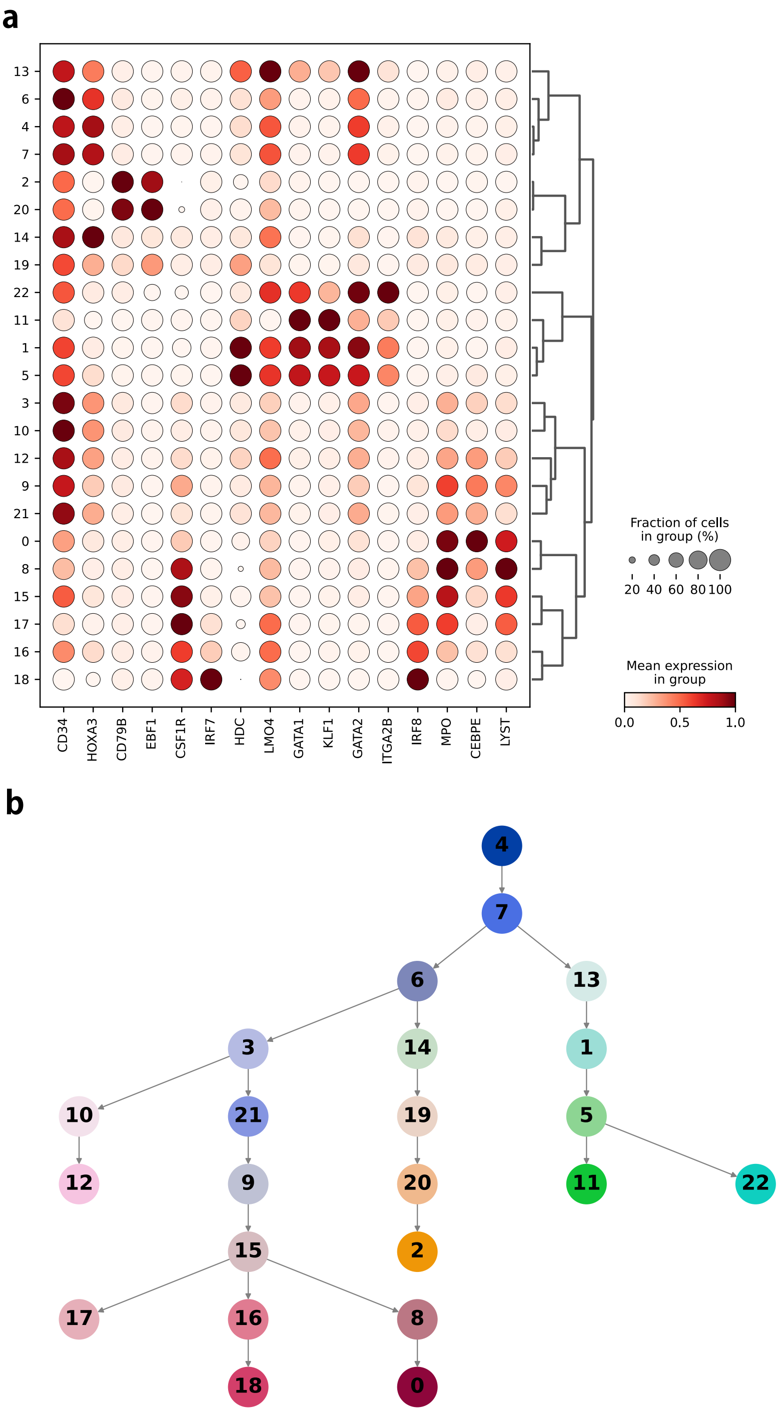

Supplementary Fig. 5 A dot plot with respect to marker genes and a trajectory tree of Setty *et al.*'s data. **a,** A dot plot of scaled expression levels of marker genes across clusters for Setty *et al.*'s data. The number appearing on the left side in the plot corresponds to the node number shown in b. **b,** A trajectory tree of Setty *et al.*'s data predicted by CAPITAL. The number in a node in the tree is equivalent to the cluster number of the same color shown in Fig. 5a in the main text.

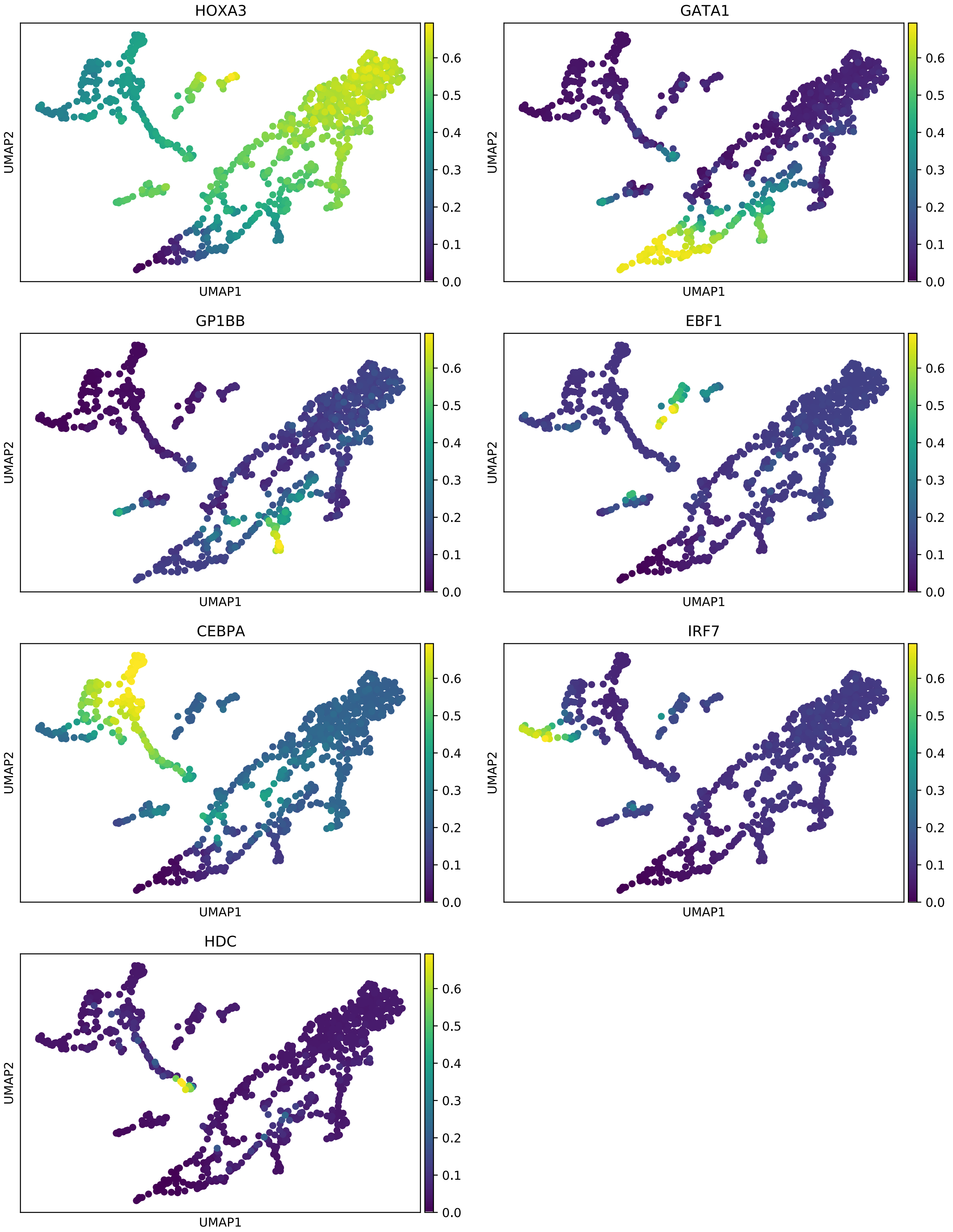

Supplementary Fig. 6 UMAP plots of cells colored by expression levels of marker genes for Velten *et al.*'s data. Selection of marker genes used in this work is based on the literature [3]. UMAP, uniform manifold approximation and projection.

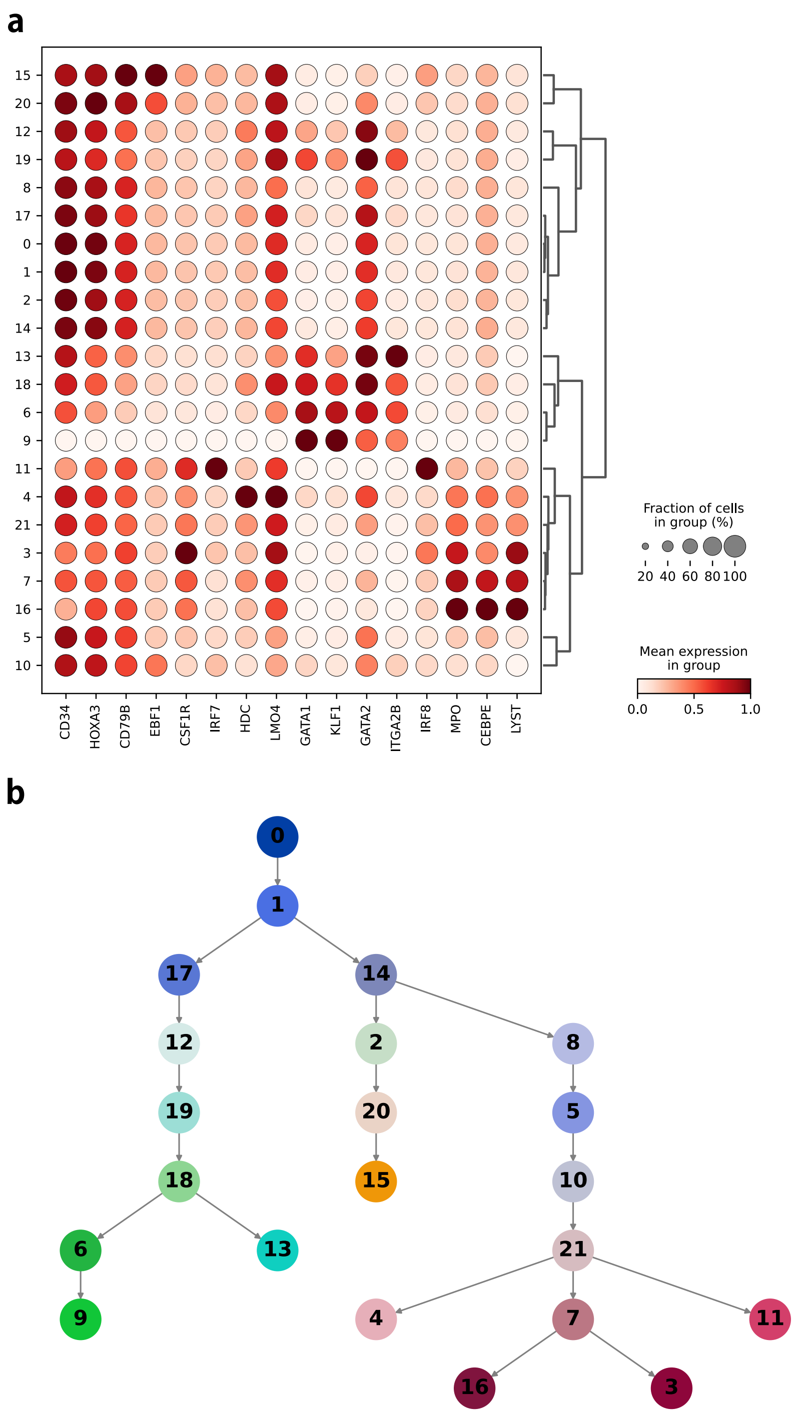

Supplementary Fig. 7 A dot plot with respect to marker genes and a trajectory tree of Velten *et al.*'s data. **a,** A dot plot of scaled expression levels of marker genes across clusters for Velten *et al.*'s data. The number appearing on the left side in the plot corresponds to the node number shown in b. **b,** A trajectory tree of Velten *et al.*'s data predicted by CAPITAL. The number in a node in the tree is equivalent to the cluster number of the same color shown in Fig. 5b in the main text.

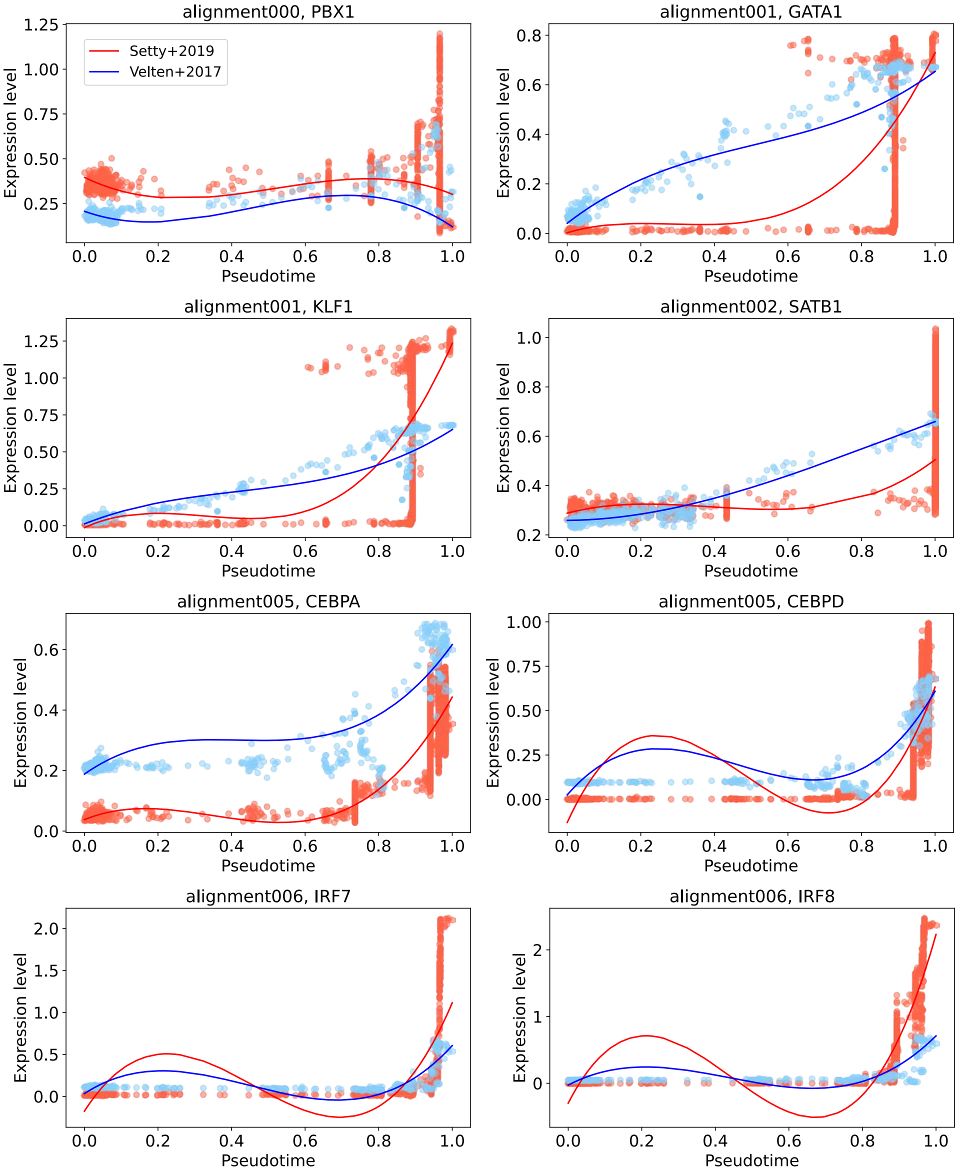

Supplementary Fig. 8 Pseudotime aligned kinetics for several markers along the paths obtained from the aligned trajectories in the human bone marrow cell datasets. alignment000, (4/HSC, 0/HSC)→(22/Mega, 13/Mega); alignment001, (4/HSC, 0/HSC)→(11/Ery, 9/Ery); alignment002, (4/HSC, 0/HSC)→(2/CLP, 15/Pre-B); alignment005, (4/HSC, 0/HSC)→(0/Mono, 3/Neutro); alignment006, (4/HSC, 0/HSC)→(18/DC, 11/Mono/DC); HSC, hematopoietic stem cell; CLP, common lymphoid progenitor; DC, dendritic cell; Ery, erythrocyte; Mega, megakaryocyte; Mono, monocyte; Neutro, neutrophil.

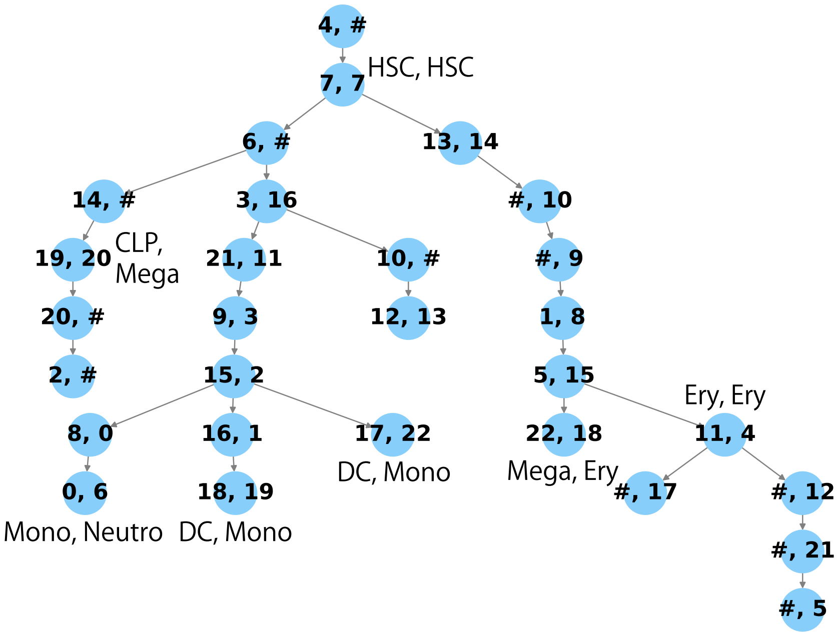

Supplementary Fig. 9 An alignment of the trajectory trees of Setty *et al.*'s data and Paul *et al.*'s data, where each pair of numbers in a node denotes the clusters shown in Fig. 5a and Fig. 6a. We should not overlook that the difference (3,109) in the number of cells in Setty(5,780)–Paul(2,671) datasets is about 1.8 times as large as that (1,756) in Velten(915)–Paul(2,671) datasets, where each number in parentheses that follows the author name shows the number of cells in that dataset. Given that both the numbers of clusters in the two datasets are the same (Figs. 5a and 6a), each cluster centroid in Paul *et al.*'s data would have less information of gene expression than in Setty *et al.*'s data, which might result in the unsuccessful alignment. # denotes a space. HSC, hematopoietic stem cell; CLP, common lymphoid progenitor; DC, dendritic cell; Ery, erythrocyte; Mega, megakaryocyte; Mono, monocyte; Neutro, neutrophil.

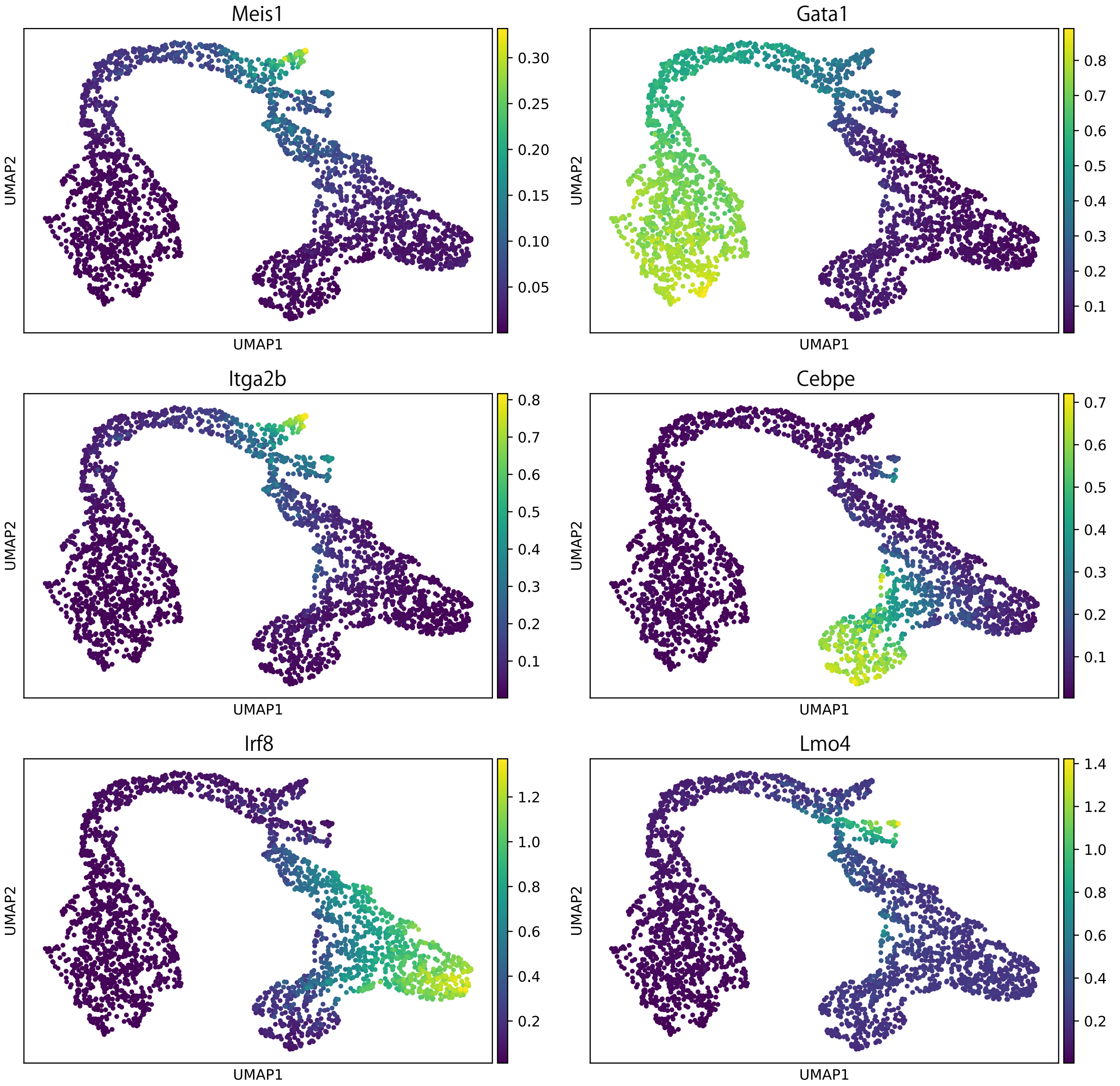

Supplementary Fig. 10 UMAP plots of cells colored by expression levels of marker genes for Paul *et al.*'s data. Selection of marker genes used in this work is based on the literature [4]. UMAP, uniform manifold approximation and projection.

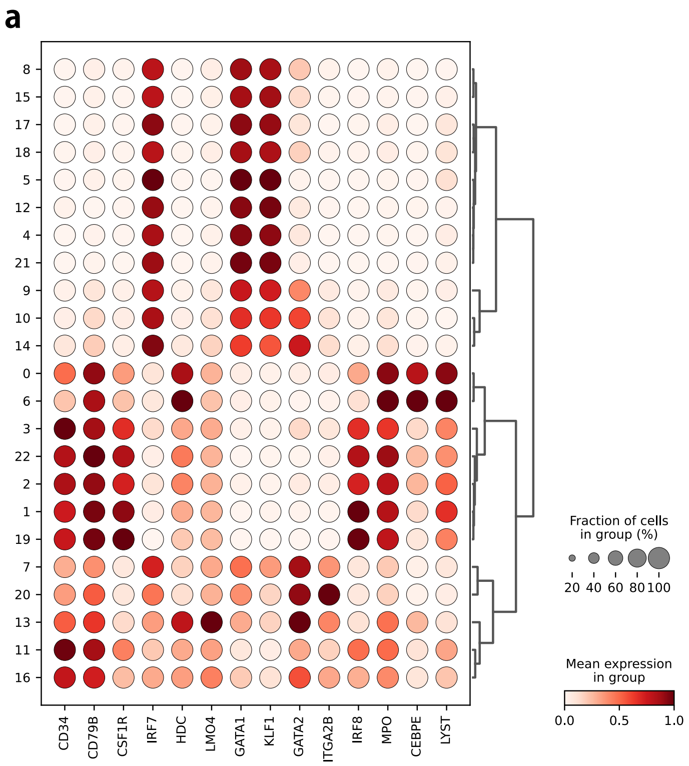

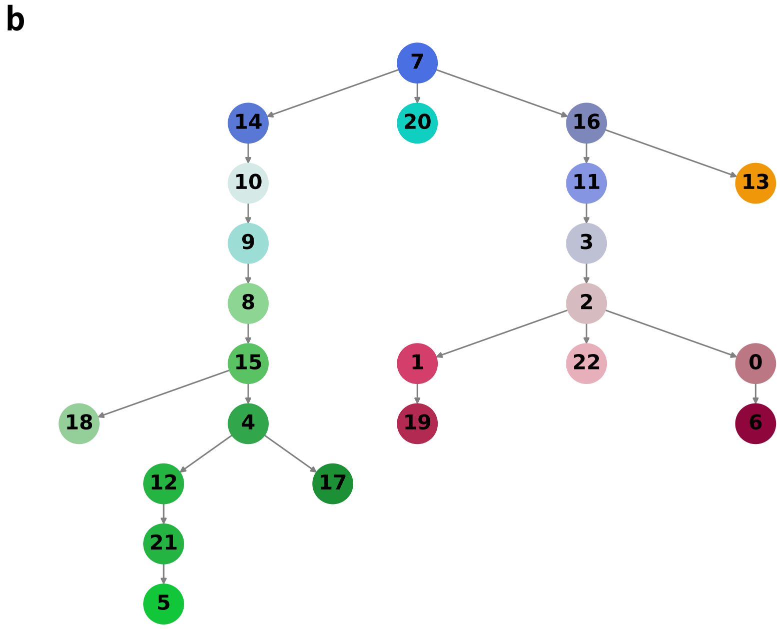

Supplementary Fig. 11 A dot plot with respect to marker genes and a trajectory tree of Paul *et al.*'s data. **a,** A dot plot of scaled expression levels of marker genes across clusters for Paul *et al.*'s data. The number appearing on the left side in the plot corresponds to the node number shown in b. **b,** A trajectory tree of Paul *et al.*'s data predicted by CAPITAL. The number in a node in the tree is equivalent to the cluster number of the same color shown in Fig. 6a in the main text.

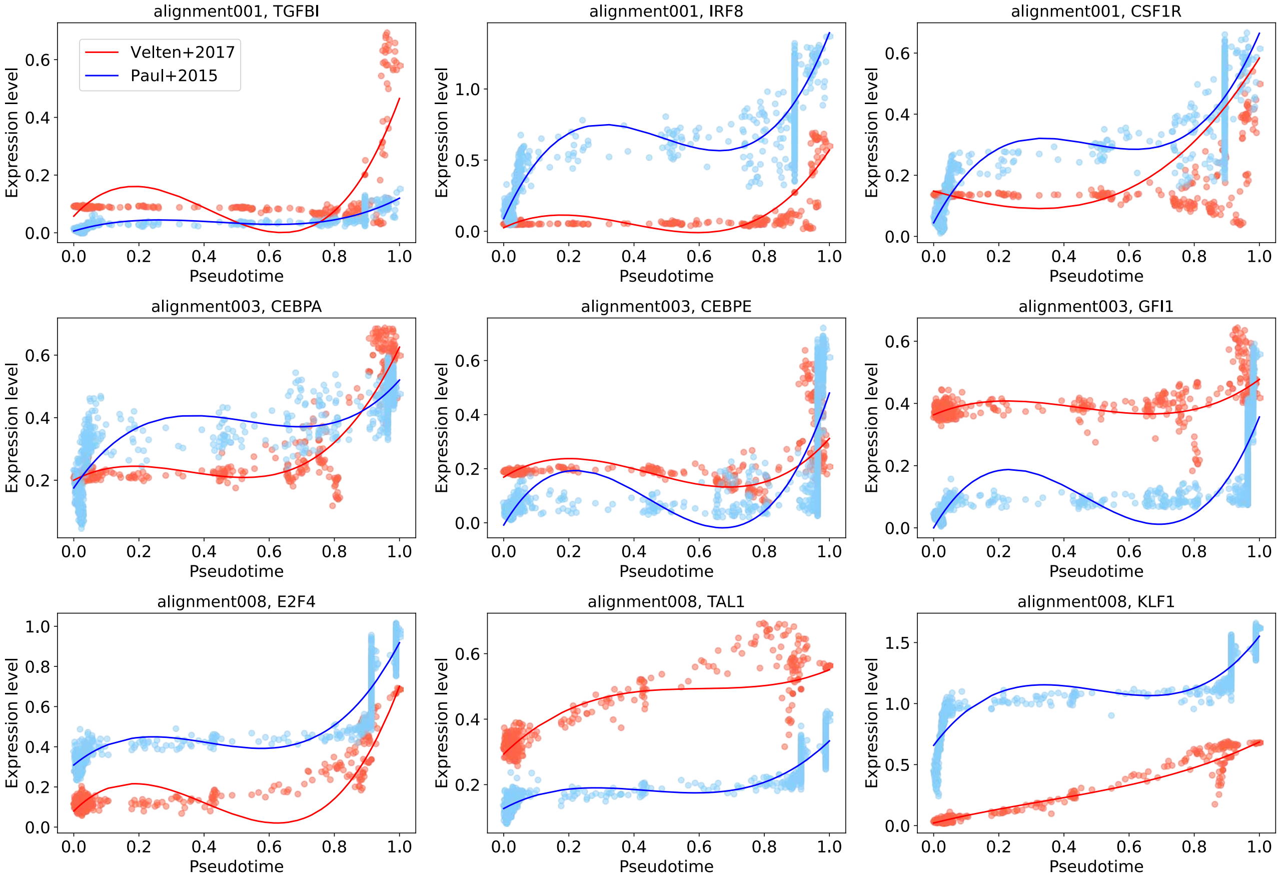

Supplementary Fig. 12 Pseudotime aligned kinetics for genes with similar patterns along the paths obtained from the aligned trajectories in the human–mouse bone marrow cell datasets. These are a set of genes that show an increasing tendency of expression both in the human and mouse datasets. alignment001, (0/HSC, 7/HSC)→(11/Mono, 19/Mono); alignment003, (0/HSC, 7/HSC)→(3/Neutro, 6/Neutro); alignment008, (0/HSC, 7/HSC)→(9/Ery, 5/Ery); HSC, hematopoietic stem cell; Ery, erythrocyte; Mono, monocyte; Neutro, neutrophil.

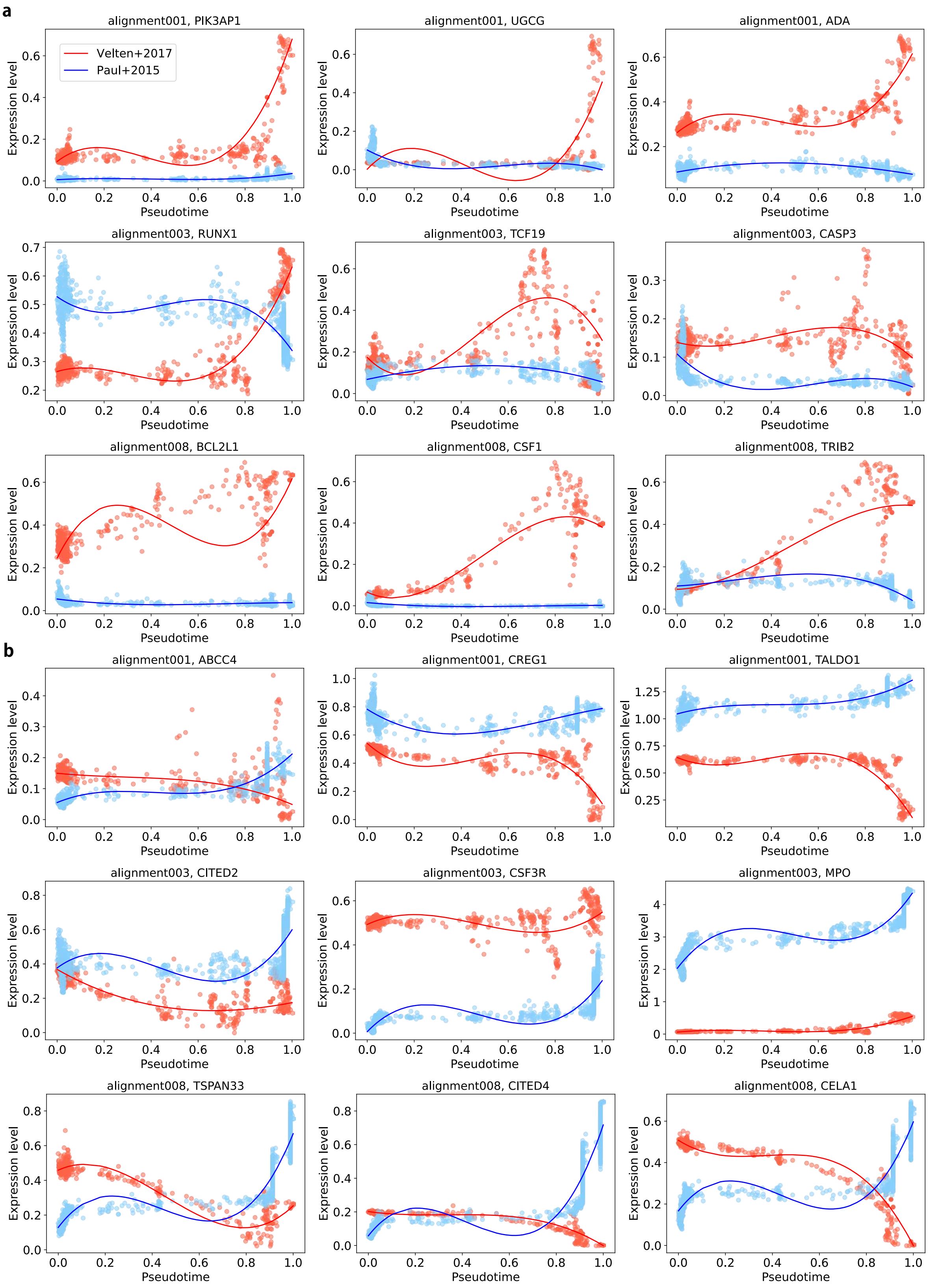
a
alignment001, PIK3AP1
Velten+2017
Paul+2015
alignment001, UGCG
alignment001, ADA
alignment003, RUNX1
alignment003, TCF19
alignment003, CASP3
alignment008, BCL2L1
alignment008, CSF1
alignment008, TRIB2
b
alignment001, ABCC4
alignment001, CREG1
alignment001, TALDO1
alignment003, CITED2
alignment003, CSF3R
alignment003, MPO
alignment008, TSPAN33
alignment008, CITED4
alignment008, CELA1
Expression level
Pseudotime

Supplementary Fig. 13 Pseudotime aligned kinetics for genes with different patterns along the paths obtained from the aligned trajectories in the human–mouse bone marrow cell datasets. **a,** These are a set of genes that show an increasing tendency of expression in the human dataset and an decreasing tendency in the mouse dataset, and **b,** vice versa. Each linear alignment is the same as Supplementary Fig. 12.

a

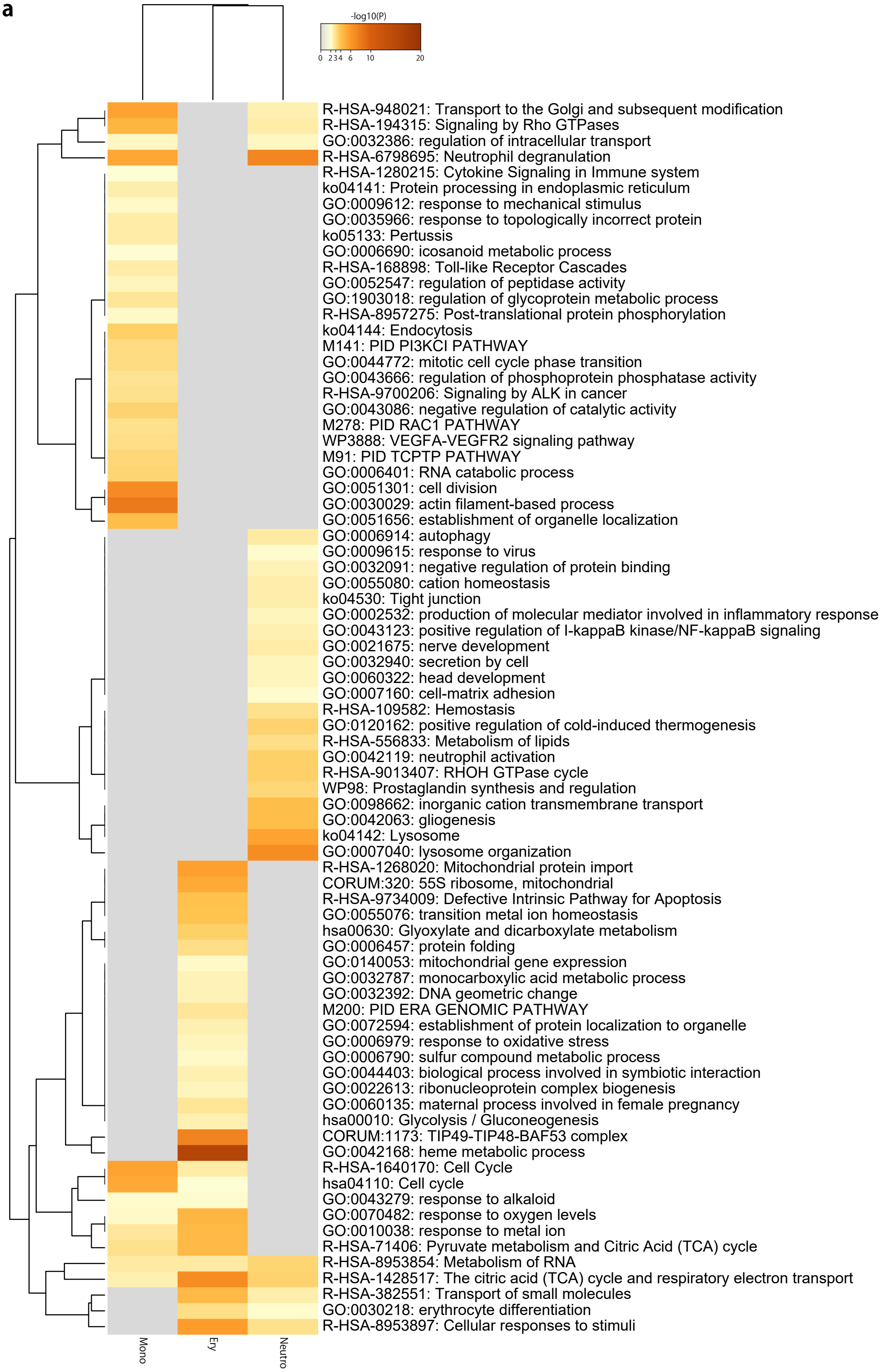
-log10(P)
0 2 3 4 6 10 20
R-HSA-948021: Transport to the Golgi and subsequent modification
R-HSA-194315: Signaling by Rho GTPases
GO:0032386: regulation of intracellular transport
R-HSA-6798695: Neutrophil degranulation
R-HSA-1280215: Cytokine Signaling in Immune system
ko04141: Protein processing in endoplasmic reticulum
GO:0009612: response to mechanical stimulus
GO:0035966: response to topologically incorrect protein
ko05133: Pertussis
GO:0006690: icosanoid metabolic process
R-HSA-168898: Toll-like Receptor Cascades
GO:0052547: regulation of peptidase activity
GO:1903018: regulation of glycoprotein metabolic process
R-HSA-8957275: Post-translational protein phosphorylation
ko04144: Endocytosis
M141: PID PI3KCI PATHWAY
GO:0044772: mitotic cell cycle phase transition
GO:0043666: regulation of phosphoprotein phosphatase activity
R-HSA-9700206: Signaling by ALK in cancer
GO:0043086: negative regulation of catalytic activity
M278: PID RAC1 PATHWAY
WP3888: VEGFA-VEGFR2 signaling pathway
M91: PID TCPTP PATHWAY
GO:0006401: RNA catabolic process
GO:0051301: cell division
GO:0030029: actin filament-based process
GO:0051656: establishment of organelle localization
GO:0006914: autophagy
GO:0009615: response to virus
GO:0032091: negative regulation of protein binding
GO:0055080: cation homeostasis
ko04530: Tight junction
GO:0002532: production of molecular mediator involved in inflammatory response
GO:0043123: positive regulation of I-kappaB kinase/NF-kappaB signaling
GO:0021675: nerve development
GO:0032940: secretion by cell
GO:0060322: head development
GO:0007160: cell-matrix adhesion
R-HSA-109582: Hemostasis
GO:0120162: positive regulation of cold-induced thermogenesis
R-HSA-556833: Metabolism of lipids
GO:0042119: neutrophil activation
R-HSA-9013407: RHOH GTPase cycle
WP98: Prostaglandin synthesis and regulation
GO:0098662: inorganic cation transmembrane transport
GO:0042063: gliogenesis
ko04142: Lysosome
GO:0007040: lysosome organization
R-HSA-1268020: Mitochondrial protein import
CORUM:320: 55S ribosome, mitochondrial
R-HSA-9734009: Defective Intrinsic Pathway for Apoptosis
GO:0055076: transition metal ion homeostasis
hsa00630: Glyoxylate and dicarboxylate metabolism
GO:0006457: protein folding
GO:0140053: mitochondrial gene expression
GO:0032787: monocarboxylic acid metabolic process
GO:0032392: DNA geometric change
M200: PID ERA GENOMIC PATHWAY
GO:0072594: establishment of protein localization to organelle
GO:0006979: response to oxidative stress
GO:0006790: sulfur compound metabolic process
GO:0044403: biological process involved in symbiotic interaction
GO:0022613: ribonucleoprotein complex biogenesis
GO:0060135: maternal process involved in female pregnancy
hsa00010: Glycolysis / Gluconeogenesis
CORUM:1173: TIP49-TIP48-BAF53 complex
GO:0042168: heme metabolic process
R-HSA-1640170: Cell Cycle
hsa04110: Cell cycle
GO:0043279: response to alkaloid
GO:0070482: response to oxygen levels
GO:0010038: response to metal ion
R-HSA-71406: Pyruvate metabolism and Citric Acid (TCA) cycle
R-HSA-8953854: Metabolism of RNA
R-HSA-1428517: The citric acid (TCA) cycle and respiratory electron transport
R-HSA-382551: Transport of small molecules
GO:0030218: erythrocyte differentiation
R-HSA-8953897: Cellular responses to stimuli
Mono
Ery
Neutro

b

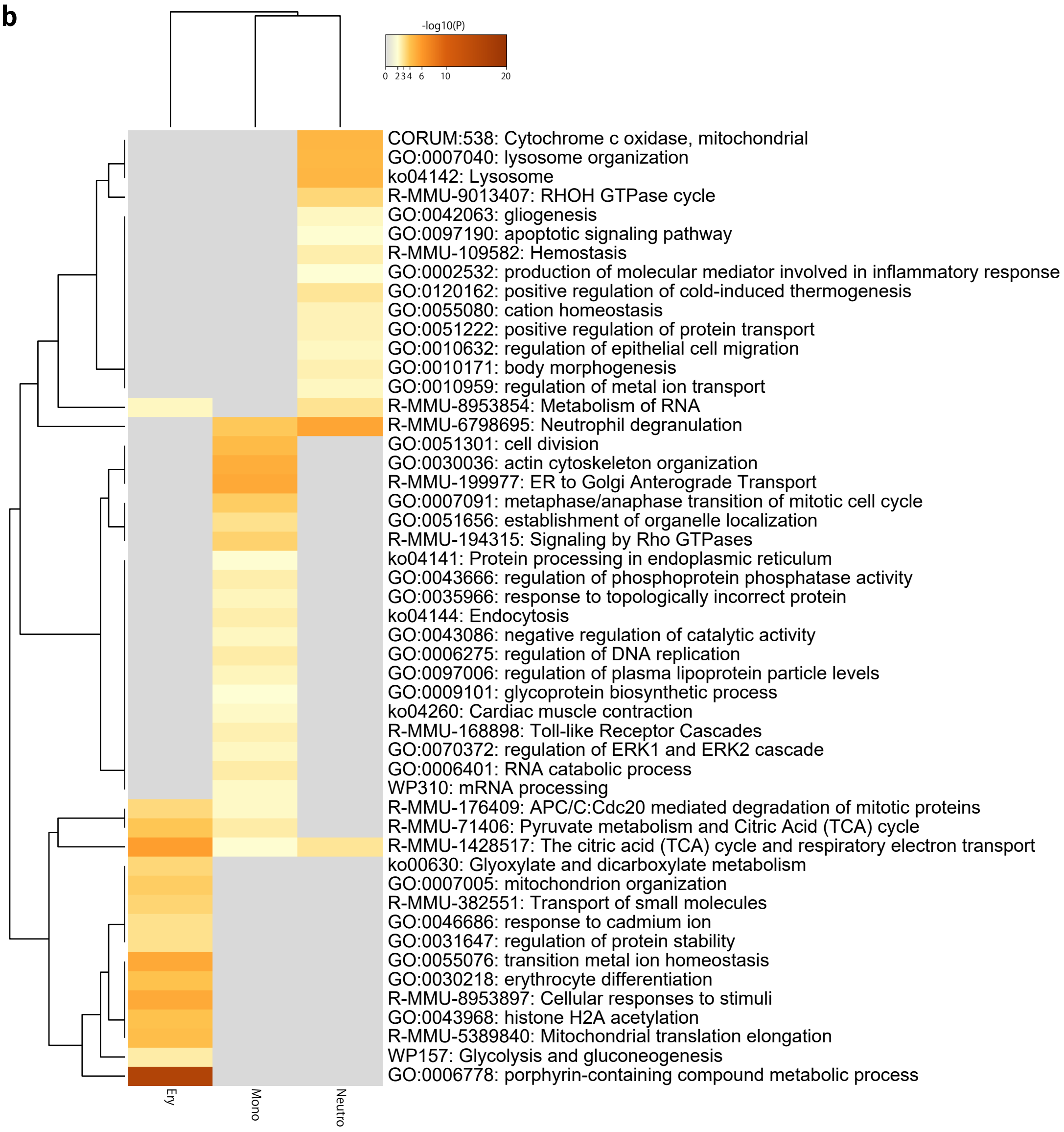
-log10(P)
0 2 3 4 6 10 20
CORUM:538: Cytochrome c oxidase, mitochondrial
GO:0007040: lysosome organization
ko04142: Lysosome
R-MMU-9013407: RHOH GTPase cycle
GO:0042063: gliogenesis
GO:0097190: apoptotic signaling pathway
R-MMU-109582: Hemostasis
GO:0002532: production of molecular mediator involved in inflammatory response
GO:0120162: positive regulation of cold-induced thermogenesis
GO:0055080: cation homeostasis
GO:0051222: positive regulation of protein transport
GO:0010632: regulation of epithelial cell migration
GO:0010171: body morphogenesis
GO:0010959: regulation of metal ion transport
R-MMU-8953854: Metabolism of RNA
R-MMU-6798695: Neutrophil degranulation
GO:0051301: cell division
GO:0030036: actin cytoskeleton organization
R-MMU-199977: ER to Golgi Anterograde Transport
GO:0007091: metaphase/anaphase transition of mitotic cell cycle
GO:0051656: establishment of organelle localization
R-MMU-194315: Signaling by Rho GTPases
ko04141: Protein processing in endoplasmic reticulum
GO:0043666: regulation of phosphoprotein phosphatase activity
GO:0035966: response to topologically incorrect protein
ko04144: Endocytosis
GO:0043086: negative regulation of catalytic activity
GO:0006275: regulation of DNA replication
GO:0097006: regulation of plasma lipoprotein particle levels
GO:0009101: glycoprotein biosynthetic process
ko04260: Cardiac muscle contraction
R-MMU-168898: Toll-like Receptor Cascades
GO:0070372: regulation of ERK1 and ERK2 cascade
GO:0006401: RNA catabolic process
WP310: mRNA processing
R-MMU-176409: APC/C:Cdc20 mediated degradation of mitotic proteins
R-MMU-71406: Pyruvate metabolism and Citric Acid (TCA) cycle
R-MMU-1428517: The citric acid (TCA) cycle and respiratory electron transport
ko00630: Glyoxylate and dicarboxylate metabolism
GO:0007005: mitochondrion organization
R-MMU-382551: Transport of small molecules
GO:0046686: response to cadmium ion
GO:0031647: regulation of protein stability
GO:0055076: transition metal ion homeostasis
GO:0030218: erythrocyte differentiation
R-MMU-8953897: Cellular responses to stimuli
GO:0043968: histone H2A acetylation
R-MMU-5389840: Mitochondrial translation elongation
WP157: Glycolysis and gluconeogenesis
GO:0006778: porphyrin-containing compound metabolic process
Ery
Mono
Neutro

Supplementary Fig. 14 Heatmaps of enriched ontology terms across genes with similar patterns along pseudotime between human and mouse bone marrow cells. They are colored by p-values computed by Metascape [5], where the one-sided statistical tests based on the hypergeometric distribution were performed. The terms for Ery, Mono and Neutro are derived from the genes that show an increasing tendency of expression for the respective alignment paths both in the human and mouse datasets. **a,** Human enriched clusters. **b,** Mouse enriched clusters. Ery, erythrocyte; Mono, monocyte; Neutro, neutrophil.

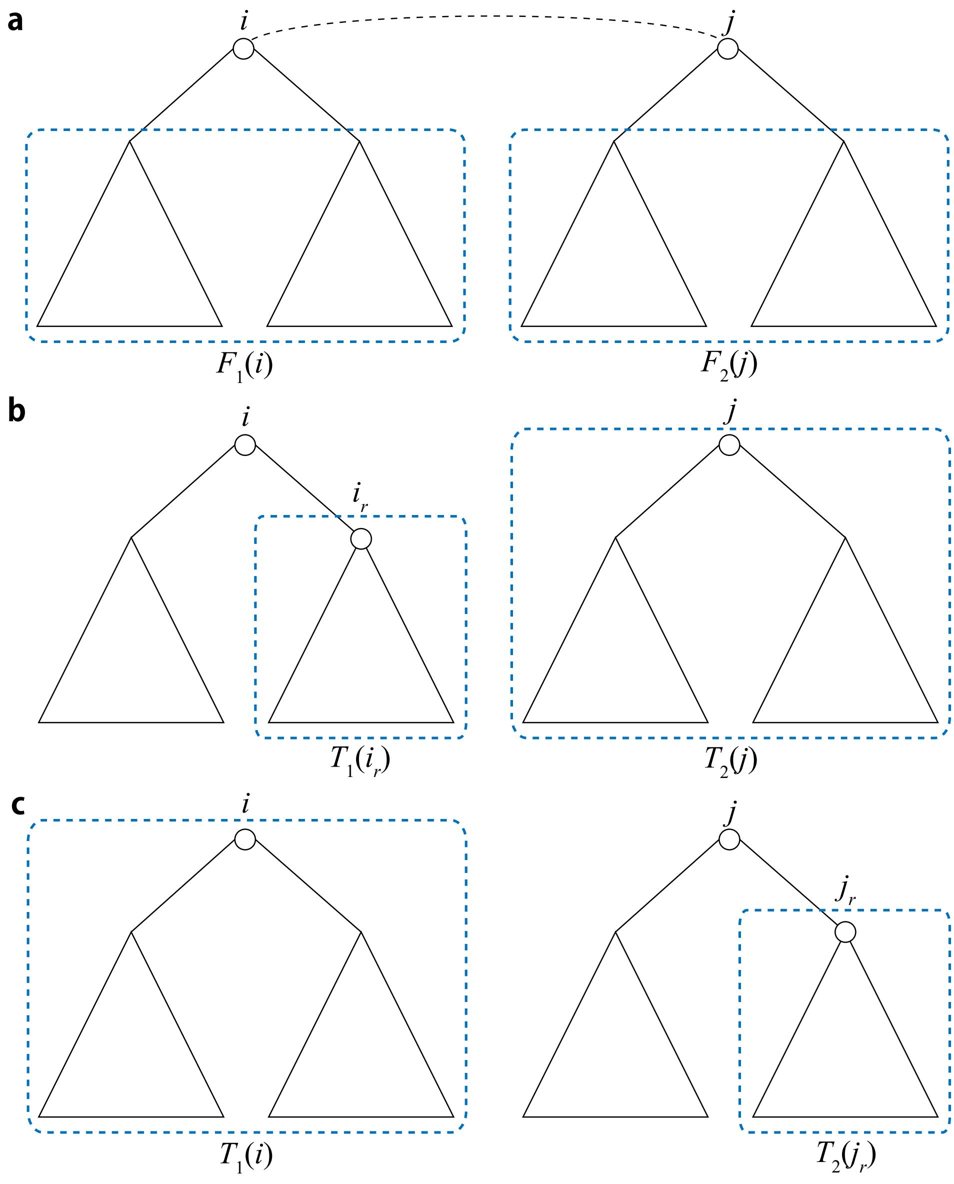

Supplementary Fig. 15 A schematic of the dynamic programming (DP) recursion for aligning trees shown in Eq. (7) in the main text. For simplicity, an example of computing a distance between only binary trees is shown. **a,** The case where nodes $i \in V(T_1)$ and $j \in V(T_2)$ are matched. A black dashed curve connects matching nodes across the trees. **b,** The case where node $i \in V(T_1)$ has no matching node in tree $T_2(j)$. **c,** The case where node $j \in V(T_2)$ has no matching node in tree $T_1(i)$.

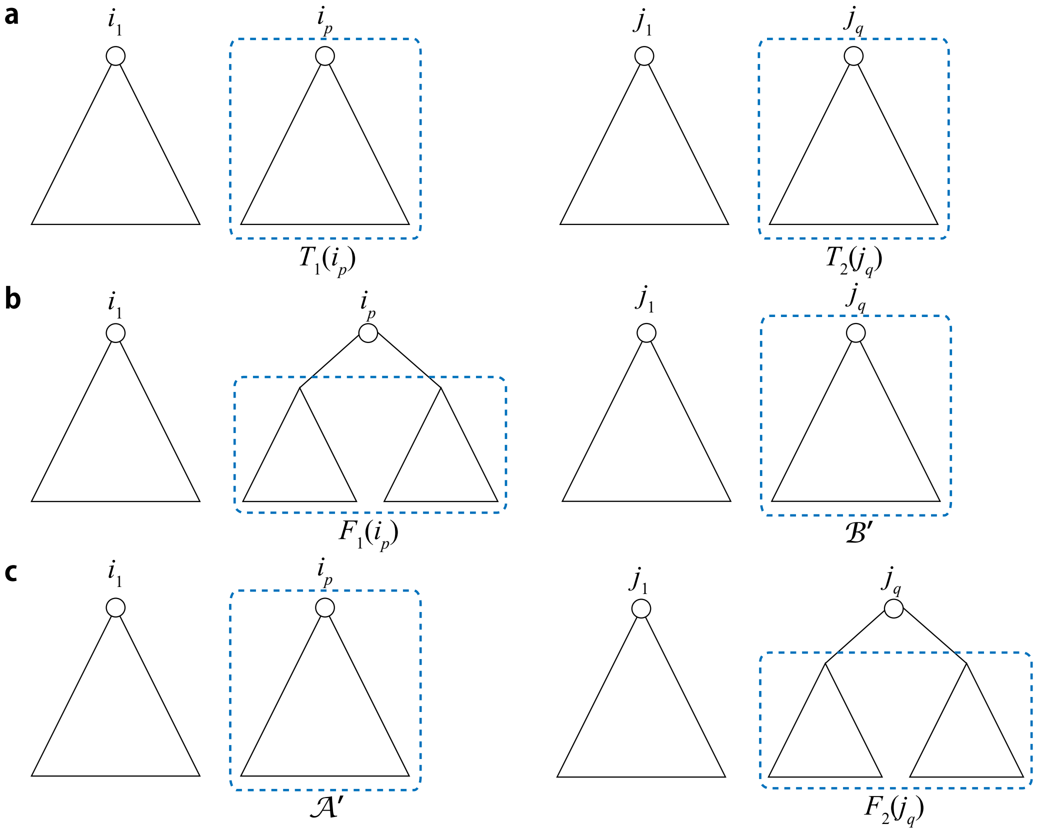

Supplementary Fig. 16 A schematic of the DP recursion for aligning forests shown in Eq. (8) in the main text. For simplicity, an example of computing a distance between forests $\mathcal{A} = \{T_1(i_1), T_1(i_2)\}$ and $\mathcal{B} = \{T_2(j_1), T_2(j_2)\}$, i.e. consisting of only binary trees, is shown. **a,** The case where all forests and trees across two inputs are matched. **b,** The case where $\mathcal{B}' = \{T_2(j_q)\}$ (e.g. $q = 2$), and node $i_p \in V(T_1)$ (e.g. $p = 2$) has no matching node in forest $\mathcal{B}$. **c,** The case where $\mathcal{A}' = \{T_1(i_p)\}$ (e.g. $p = 2$), and node $j_q \in V(T_2)$ (e.g. $q = 2$) has no matching node in forest $\mathcal{A}$.

References

[1] Haghverdi, L., Büttner, M., Wolf, F. A., Buettner, F. & Theis, F. J. Diffusion pseudotime robustly reconstructs lineage branching. *Nat. Methods* **13**, 845–848 (2016).

[2] Setty, M. *et al.* Characterization of cell fate probabilities in single-cell data with Palantir. *Nat. Biotechnol.* **37**, 451–460 (2019).

[3] Velten, L. *et al.* Human haematopoietic stem cell lineage commitment is a continuous process. *Nat. Cell Biol.* **19**, 271–281 (2017).

[4] Paul, F. *et al.* Transcriptional heterogeneity and lineage commitment in myeloid progenitors. *Cell* **163**, 1663–1677 (2015).

[5] Zhou, Y. *et al.* Metascape provides a biologist-oriented resource for the analysis of systems-level datasets. *Nat. Commun.* **10**, 1523 (2019).
