# Supplementary material for: Alignment of single-cell trajectory trees with CAPITAL
Source: Nat Commun. 2022 Oct 14;13:5972. doi: 10.1038/s41467-022-33681-3 (PMC9568509; doi:10.1038/s41467-022-33681-3)
Supplement: Supplementary file 3 — Reporting Summary [file 41467_2022_33681_MOESM3_ESM.pdf]

nature portfolio

Corresponding author(s): Yuki Kato
NCOMMS-21-49857C

Last updated by author(s): Sep 17, 2022

Reporting Summary

Nature Portfolio wishes to improve the reproducibility of the work that we publish. This form provides structure for consistency and transparency in reporting. For further information on Nature Portfolio policies, see our Editorial Policies and the Editorial Policy Checklist.

Statistics

For all statistical analyses, confirm that the following items are present in the figure legend, table legend, main text, or Methods section.

n/a	Confirmed	
☐	☒	The exact sample size (*n*) for each experimental group/condition, given as a discrete number and unit of measurement
☐	☒	A statement on whether measurements were taken from distinct samples or whether the same sample was measured repeatedly
☐	☒	The statistical test(s) used AND whether they are one- or two-sided<br>*Only common tests should be described solely by name; describe more complex techniques in the Methods section.*
☒	☐	A description of all covariates tested
☒	☐	A description of any assumptions or corrections, such as tests of normality and adjustment for multiple comparisons
☐	☒	A full description of the statistical parameters including central tendency (e.g. means) or other basic estimates (e.g. regression coefficient) AND variation (e.g. standard deviation) or associated estimates of uncertainty (e.g. confidence intervals)
☐	☒	For null hypothesis testing, the test statistic (e.g. *F*, *t*, *r*) with confidence intervals, effect sizes, degrees of freedom and *P* value noted<br>*Give P values as exact values whenever suitable.*
☒	☐	For Bayesian analysis, information on the choice of priors and Markov chain Monte Carlo settings
☒	☐	For hierarchical and complex designs, identification of the appropriate level for tests and full reporting of outcomes
☒	☐	Estimates of effect sizes (e.g. Cohen's *d*, Pearson's *r*), indicating how they were calculated

Our web collection on statistics for biologists contains articles on many of the points above.

Software and code

Policy information about availability of computer code

Data collection	dyngen (1.0.3) in R and Scanpy (1.9.1) in Python were used.
Data analysis	Our tool CAPITAL (1.0.13) in Python that employs Scanpy (1.9.1) and MAGIC (3.0.0), and Scanorama (1.7.0) and scVI (0.16.0) in scib (1.0.3) in Python, and Seurat (4.1.1) in R were used. CAPITAL codes and documentation with tutorials are accessible through a GitHub repository at https://github.com/ykat0/capital, whose DOI is https://doi.org/10.5281/zenodo.7088325, as well as a Code Ocean compute capsule at https://codeocean.com/capsule/5673663/tree/v1.

For manuscripts utilizing custom algorithms or software that are central to the research but not yet described in published literature, software must be made available to editors and reviewers. We strongly encourage code deposition in a community repository (e.g. GitHub). See the Nature Portfolio guidelines for submitting code & software for further information.

Data

Policy information about availability of data

All manuscripts must include a data availability statement. This statement should provide the following information, where applicable:

- Accession codes, unique identifiers, or web links for publicly available datasets
- A description of any restrictions on data availability
- For clinical datasets or third party data, please ensure that the statement adheres to our policy

The original single-cell expression count dataset of human bone marrow cells provided by Setty et al. is available at https://github.com/dpeerlab/Palantir. The other human bone marrow cells dataset compiled by Velten et al. is available from Gene Expression Omnibus (GSE75478) [https://www.ncbi.nlm.nih.gov/geo/query/acc.cgi?acc=GSE75478]. The mouse dataset provided by Paul et al. is accessible when running the datasets.paul15() function in Scanpy (1.9.1). The synthetic datasets, and human and mouse cell datasets preprocessed in this study are available through a GitHub repository at https://github.com/ykat0/capital, whose DOI is

https://doi.org/10.5281/zenodo.7088325. They are also available in a Code Ocean compute capsule at https://codeocean.com/capsule/5673663/tree/v1.

Field-specific reporting

Please select the one below that is the best fit for your research. If you are not sure, read the appropriate sections before making your selection.

- [x] Life sciences
- [ ] Behavioural & social sciences
- [ ] Ecological, evolutionary & environmental sciences

For a reference copy of the document with all sections, see nature.com/documents/nr-reporting-summary-flat.pdf

Life sciences study design

All studies must disclose on these points even when the disclosure is negative.

Sample size	No statistical method was used to predetermine sample size.
Data exclusions	Some outlier cells in isolated clusters in the original public data were excluded according to pre-established criteria.
Replication	We have confirmed the same alignment results using the same preprocessed datasets on different computers and Code Ocean at least 30 times.
Randomization	No samples were allocated to experimental groups.
Blinding	There was no group allocation to which the investigators were blinded.

Reporting for specific materials, systems and methods

We require information from authors about some types of materials, experimental systems and methods used in many studies. Here, indicate whether each material, system or method listed is relevant to your study. If you are not sure if a list item applies to your research, read the appropriate section before selecting a response.

Materials & experimental systems

n/a	Involved in the study	
[x]	[ ]	Antibodies
[x]	[ ]	Eukaryotic cell lines
[x]	[ ]	Palaeontology and archaeology
[x]	[ ]	Animals and other organisms
[x]	[ ]	Human research participants
[x]	[ ]	Clinical data
[x]	[ ]	Dual use research of concern

Methods

n/a	Involved in the study	
[x]	[ ]	ChIP-seq
[x]	[ ]	Flow cytometry
[x]	[ ]	MRI-based neuroimaging
